# Downregulation of RNF138 inhibits cellular proliferation, migration, invasion and EMT in glioma cells via suppression of the Erk signaling pathway

**DOI:** 10.3892/or.2022.8337

**Published:** 2022-05-23

**Authors:** Haibin Wu, Xuetao Li, Ming Feng, Lin Yao, Zhitong Deng, Guozheng Zao, Youxin Zhou, Sansong Chen, Ziwei Du

Oncol Rep 40: 3285–3296, 2018; DOI: 10.3892/or.2018.6744

Following the publication of the above paper, during a routine examination of the raw data the authors noticed errors in Fig. 5 in the published version of the article. Essentially, in Fig. 5A on p. 3291, the western blot data for MMP2 (for the U87 cell line) did not match with the original data: An image from Fig. 7A (the western blotting data for Bcl2 in the U87 cell line had been erroneously selected during the process of assembling the figure); however, the authors were able to locate the original western blot data for MMP2 pertaining to Fig. 5A, and the corrected version of Fig. 5 is shown below.

Note that these errors did not affect the overall conclusions reported in the study. All the authors agree to the publication of this corrigendum, and are grateful to the Editor of *Oncology Reports* for allowing them the opportunity to publish it; furthermore, they apologize for any inconvenience caused to the readership of the Journal.

## Figures and Tables

**Figure 5.**
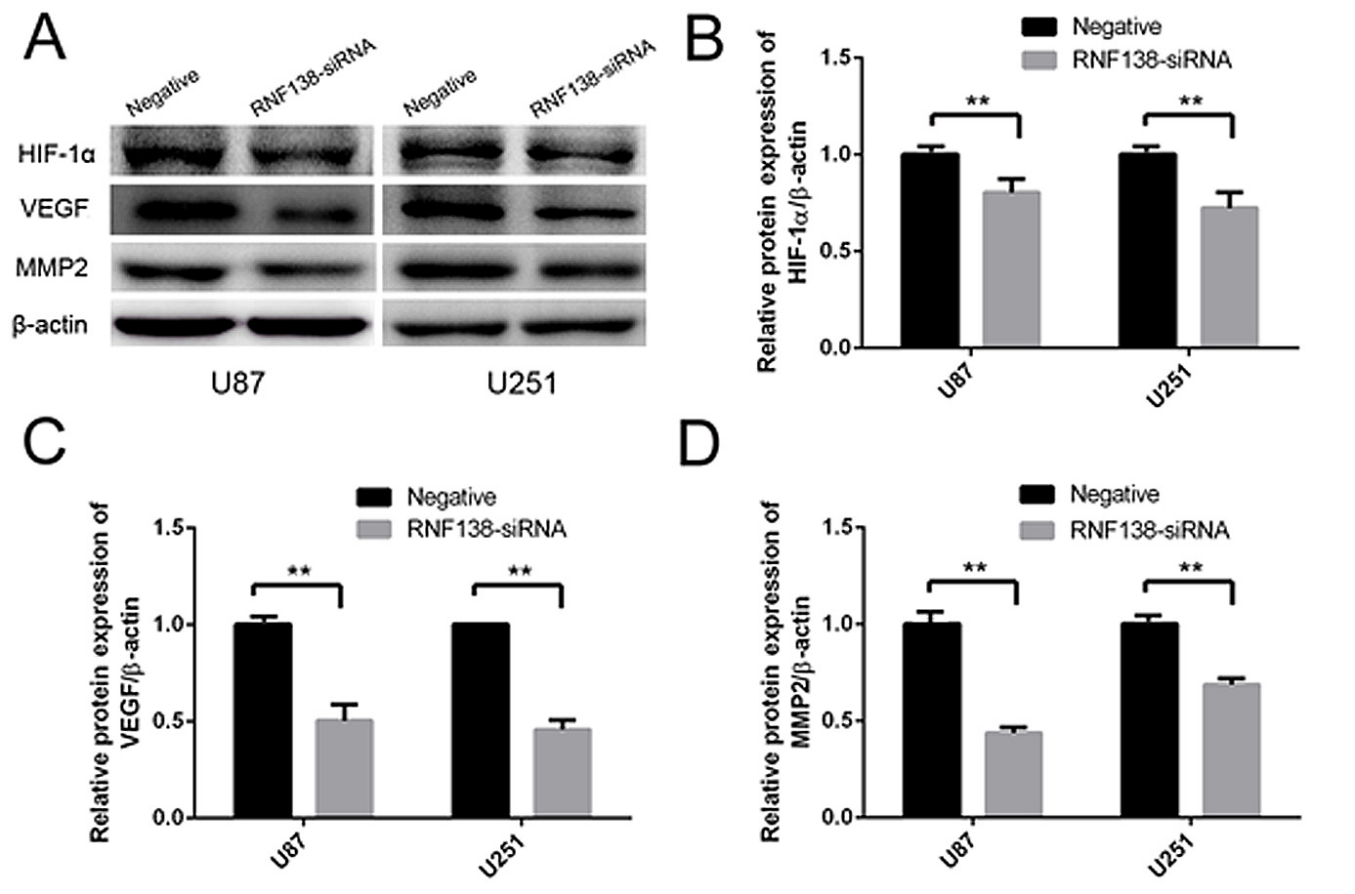
RNF138 regulates the protein levels of HIF-1α, VEGF and MMP2 in glioma cell lines. (A) Western blotting analysis of HIF-1α, VEGF and MMP2 expression in U87 and U251 cells, β-actin was used as a loading control. Densitometry analysis of (B) HIF-1α, (C) VEGF and (D) MMP2 expression in U87 and U251 cells using ImageJ analysis. Data are presented as the mean ± standard deviation for three independent experiments. **P<0.01. RNF138, ring finger protein 138; siRNA, small interfering RNA; HIF-1α, hypoxia-inducible factor-1α; VEGF, vascular endothelial growth factor; MMP2, matrix metalloproteinase 2.

